# Insights into Cancer-Associated Thrombosis Leading Towards Ischemic Stroke

**DOI:** 10.3390/biology14010050

**Published:** 2025-01-10

**Authors:** Surajit Hansda, Hiranmoy Das

**Affiliations:** Department of Pharmaceutical Sciences, Jerry H. Hodge School of Pharmacy, Texas Tech University Health Sciences Center, Amarillo, TX 79106, USA

**Keywords:** cancer-associated stroke, cancer, ischemic stroke, thrombosis, coagulopathy

## Abstract

Stroke leads to significant disability in most patients, whereas cancer elevates the occurrence of stroke. The incidence of cancer-associated stroke (CAS) is projected to rise as a result of improvements in cancer therapies. Various forms of cancer have been demonstrated to be linked to ischemic stroke. Cancer might influence stroke pathophysiology either directly or through coagulation that creates a hypercoagulative state, in addition to infections. Treatment methods for cancer, including chemotherapy, radiotherapy, and surgery, have all been demonstrated to increase the risk of stroke as well. This review discusses the subtypes, epidemiology, pathophysiology, mechanisms of stroke within cancer patients, biomarkers, and signaling pathways of stroke in cancer while providing vital information on the involved transcription factors, treatment, and management of patients with cancer-associated ischemic stroke. Atherosclerosis, extracellular vesicles (EVs), and signaling biomolecules can also affect CAS. Overall, stroke is a significant and not uncommon complication of cancer, and there is an immediate demand for neurologists and oncologists to create strategies for screening and preventing strokes in cancer patients.

## Simple Summary

Cancer increases the risk of stroke due to the increment of blood clotting caused by cancer. These clots block blood flow to the brain, causing a stroke. Additionally, cancer therapy, including chemotherapy and radiation, surgery, hormonal therapy, and central venous catheters, increases the risk. Detection of cancer-associated stroke is challenging because it happens suddenly and does not show typical stroke symptoms. Understanding the link between cancer and stroke helps to improve diagnosis, develop prevention strategies, and support patient care.

## 1. Introduction

The estimated lifetime incidence of cancer is around 40%, with around 2 million new cancer cases and 609,820 cancer deaths occurring in the United States in the year 2023 [1]. Cancer remains one of the leading causes of death worldwide, and its impact is widespread across societies, economies, and healthcare systems. The burden of cancer is rising, not only due to an aging population but also because of several factors that contribute to cancer’s growing impact, including lifestyle factors, environmental exposures, and the increasing complexity of the disease itself. Cancer treatment faces several significant challenges, such as tumor heterogeneity, resistance to treatment, early detection and diagnosis, side effects of treatment, immunotherapy complications, etc., that impact its effectiveness, accessibility, and outcomes. Therefore, long-term quality of life has become more important for cancer patients, making the prevention of diseases and cancer-related complications diverse, which can arise both from the disease itself and as a result of its treatment. These complications can significantly impact the patient’s quality of life, prognosis, and survival. They may involve the cancer directly (such as tumor growth affecting nearby structures) or be secondary to surgery, chemotherapy, radiation therapy, or immunotherapy, which are used for the treatment of cancer. Some complications are acute, while others are chronic and long-lasting. Cancer-related complications are wide-ranging, affecting nearly every organ system, and they can be caused by the cancer itself, its treatments, or a combination of both.

Stroke is the third leading cause of death and disability among Americans [2]. The cause of stroke has a wide range of etiologies and can occur due to various underlying conditions affecting different parts of the brain. Strokes are typically classified into two main types based on their cause: ischemic stroke (caused by a blockage of or reduction in blood flow) and hemorrhagic stroke (caused by bleeding in or around the brain). Each type of stroke has its own set of etiologies, but many of the risk factors overlap. An ischemic stroke occurs when blood flow to a part of the brain is blocked or reduced, depriving brain tissue of oxygen and nutrients. This can result from a blockage in a cerebral artery due to a thrombosis (blood clot) or an embolism (a clot or plaque fragment traveling from another part of the body). Embolic and thrombotic strokes are ischemic strokes, which occur when a blood clot blocks blood flow to the brain. A blood clot or plaque breaks off from somewhere else in the body, such as the heart or neck, and travels to the brain.

Cancer patients frequently develop cerebrovascular illnesses, and there have been reports of a link between cancer and stroke. According to nationwide studies conducted in the US, Europe, and Asia, the risk of ischemic stroke rises in the initial months (up to a year) following a cancer diagnosis. Since ancient times, doctors have understood that cancer raises the risk of venous thromboembolism and that the majority of thrombotic events in cancer patients occur in the venous circulation. Apart from patent foramen ovale (PFO), nonstenosing big-artery atherosclerosis, and left-atrial cardiopathy, malignancy is a significant and prevalent subtype of ischemic stroke [3,4]. Approximately 10% of patients with ischemic stroke from all causes have known malignancy, according to claims-based data from the *National Inpatient Sample* [5]. One known risk factor for ischemic stroke is active malignancy. The risk of ischemic stroke increased by 59% in the year before a cancer diagnosis, according to a comprehensive study that used data from the *American Cancer Registry* connected to *Medicare* claims [6]. Physicians are now paying more attention to ischemic stroke in cancer patients as a result of these new results, particularly since arterial occurrences typically have a greater impact than venous systems. The rapidly expanding subspecialty of “oncocardiology” is indicative of this greater focus among cardiologists. However, neurologists have also become more conscious of this increasingly acknowledged clinical issue. The underlying processes and suitable therapeutic approaches are still unknown and unclear despite the body of evidence regarding the correlation between cancer and stroke. The absence of high-quality data on the best practices for cancer patients’ acute therapy and stroke prevention goes hand in hand with this mechanistic uncertainty. Studying cancer-associated stroke (CAS) is important for several reasons, as it contributes to a better understanding of the complex relationship between cancer and stroke, informs current clinical practice, and guides future research endeavors. This is critical due to the increasing incidence of both cancer and stroke, the complexity of managing cancer patients with elevated stroke risk, and the significant impact strokes can have on cancer survival patients and the quality of life of the patients. Herein, we will discuss the possibility of cancer-related ischemic stroke and critically evaluate newly discovered evidence that connects cancer to ischemic stroke, emphasizing causes, biomarkers, outcomes, management, existing knowledge gaps, and possible research approaches to fill them.

## 2. Cancer-Associated Thrombosis

Thromboembolism refers to forming a blood clot (thrombus) that breaks loose and travels through the bloodstream, potentially lodging in a distant vessel and causing a blockage. The term “thromboembolism” combines thrombosis (clot formation) and embolism (obstruction by a foreign substance, in this case, a blood clot). Thromboembolism in cancer is a significant and often overlooked complication of malignancy. Cancer patients have a higher risk of developing blood clots, which can lead to venous thromboembolism (VTE), including deep vein thrombosis (DVT) and pulmonary embolism (PE). Cancer is highly thrombogenic and increases the risk of arterial and venous thromboembolism [7]. As early as 1823, it was reported that thrombosis and cancer were related. VTE is very common in patients with cancer, with a 20% prevalence [8]. Additionally, one study discovered that cancer patients who experienced arterial thromboembolism had a poor prognosis and a three-fold-higher risk of dying [9]. Thromboembolic events are one of the leading causes of morbidity and mortality in cancer patients, and their occurrence can complicate cancer treatment and affect outcomes. There are mainly two types of thromboembolic events in cancer: VTE and arterial thromboembolism (ATE). VTE is the most common type of thromboembolic event in cancer patients and includes DVT and PE. DVT occurs when a blood clot forms in the deep veins, typically in the legs, and can travel to the lungs, causing PE, a life-threatening condition. VTE is up to seven times more common in cancer patients compared to the general population. Arterial thromboembolism includes conditions like stroke (often embolic), myocardial infarction (heart attack), and peripheral artery occlusion. ATE events are less common in cancer patients compared to VTE but are still significant, particularly in cancers that affect the heart, lungs, or those with associated comorbidities (e.g., atrial fibrillation) [10].

Cancer-associated thrombosis is distinct from general thrombosis due to the presence of specific risk factors associated with cancer. Several mechanisms explain why cancer patients are at higher risk of thrombosis. Cancer triggers a hypercoagulable state (an increased tendency for blood to clot), which is one of the most significant factors for thromboembolism in cancer patients. Tumor cells produce procoagulant substances, such as tissue factor and cancer procoagulant, that activate the clotting cascade [11]. Additionally, inflammatory cytokines like interleukin-6 (IL-6) and tumor necrosis factor-alpha (TNF-α), released by both the tumor and the immune response, contribute to increased clot formation. Cytokine storm in some cancers, such as pancreatic cancer or lung cancer, further enhances the pro-thrombotic environment. Another important mechanism is endothelial injury, where tumors can directly damage blood vessel walls (endothelium), leading to endothelial dysfunction. This can promote clot formation by exposing underlying tissue factors and impairing normal blood flow. Some cancer treatments, such as chemotherapy and radiation therapy, can also cause endothelial injury, further increasing the risk of thrombosis. Tumors can lead to blood stasis, particularly in veins, by compressing blood vessels or obstructing blood flow. This can increase the risk of clot formation [12,13]. Additionally, certain cancers (e.g., multiple myeloma or leukemias) can increase blood viscosity, making clot formation more likely. Cancer patients who are undergoing surgery, particularly those with advanced disease or who are immobile due to pain or weakness, are at heightened risk for VTE due to stasis of blood flow in veins. Cancer patients, especially those who are receiving cancer treatments such as chemotherapy, radiation therapy, hormonal, or immunotherapy, have a much higher risk of DVT than other people. Several chemotherapeutic agents, including cisplatin, gemcitabine, and doxorubicin, can increase the risk of thrombosis by inducing endothelial damage, inflammation, and hypercoagulability. Radiation can damage blood vessels and increase the likelihood of thrombosis. Some hormonal therapies (e.g., tamoxifen used in breast cancer) are associated with an increased risk of VTE, particularly in patients with other risk factors. Newer cancer treatments, including immune checkpoint inhibitors, can increase the risk of thrombosis due to immune-mediated mechanisms that promote clotting. While some cancer cells can produce cancer procoagulant, which acts directly on Factor Xa, tissue factor, which is produced by malignant cells, seems to set off the coagulation cascade that results in the formation of Factor Xa. Thromboembolic events can be the initial symptom, or they can happen to patients who have a known history of cancer. In patients with venous thromboembolism, arterial thromboembolism with ischemic stroke and myocardial infarction is less common than deep vein thrombosis or pulmonary embolism [14]. Heritable thrombophilia, which includes genetic predispositions to blood clotting disorders, has been studied in the context of cancer for its potential role in increasing the risk of VTE, and thrombotic conditions, such as the factor V Leiden mutation, prothrombin G20210A mutation, and deficiencies in antithrombin, protein C, or protein S, may further amplify this risk. The direct association of VTE with cancer development is yet to be defined. Research on whether heritable thrombophilia predisposes individuals to cancer remains inconclusive, with no strong evidence linking these genetic conditions to increased cancer incidence yet [15,16].

Certain cancers (pancreatic, lung, and breast cancer, etc.) have a particularly strong association with thromboembolism due to the specific biological features of the tumor, the treatments used, and the hypercoagulable state they induce [17]. Pancreatic cancer is one of the most strongly associated cancers with VTE and other thromboembolic events. Patients with pancreatic cancer have a significantly higher risk of developing both DVT and PE. The tumor’s procoagulant activity, along with the inflammatory response, contributes to this high risk. Non-small-cell lung cancer (NSCLC) and small-cell lung cancer (SCLC) are also frequently associated with thrombosis. Patients with advanced lung cancer often have elevated levels of inflammatory markers and cytokines, contributing to a hypercoagulable state. Additionally, lung cancer patients may experience atrial fibrillation, a risk factor for arterial thromboembolism. Colorectal cancer and gastric cancer are also associated with an increased risk of VTE. The risk is particularly high in patients with advanced or metastatic disease. Leukemias (especially acute myeloid leukemia (AML)), lymphomas, and multiple myeloma carry a higher risk of thrombosis due to factors like high blood cell counts, endothelial damage, and treatment-related risks. The use of induction chemotherapy in leukemia patients is particularly associated with a high incidence of thrombosis. Although less often associated with thromboembolism compared to other cancers, breast cancer, when advanced or treated with tamoxifen, can increase the risk of VTE. Cancer patients have additional risk factors that may contribute to thrombosis. The later the stage, the higher the risk of thrombosis, and post-surgical patients or those who are immobile due to cancer or treatment are at increased risk. Older cancer patients are more likely to develop thromboembolism. Conditions like hypertension, diabetes, heart disease, and obesity can further elevate thromboembolic risk in cancer patients. Chemotherapy, radiation, and immunotherapy increase the risk of thrombosis [18,19,20].

Cancer and thromboembolism are intricately linked, with cancer patients facing a significantly higher risk of both venous and arterial thromboembolic events due to the hypercoagulable state associated with malignancy, cancer treatments, and other comorbid conditions. Preventive and therapeutic strategies, including anticoagulation therapy and careful management of cancer treatments, are essential for reducing the risk of thromboembolism in this high-risk population. Early detection and intervention are key to improving outcomes in cancer patients with thromboembolic complications.

## 3. Cancer and Embolic Stroke

Cancer can directly or indirectly contribute to an embolic stroke. Cancerous tumors, particularly those in the lungs, prostate, and pancreas, during metastasis, can release cells into the bloodstream, which may embolize and block blood vessels in the brain. People with cancer are at increased risk of developing blood clots, which can travel (embolize) from other parts of the body, such as the heart or veins, and cause an embolic stroke [21]. Some cancers, like lung cancer or leukemia, can increase the risk of heart-related complications, such as atrial fibrillation or endocarditis, which can also lead to embolic strokes. Cancers, like pancreatic, lung, and brain, are more strongly associated with an increased risk of embolic stroke. Chemotherapy and radiation therapy, particularly in the chest or head/neck areas, can affect blood clotting, vascular health, and blood flow, all of which can contribute to stroke risk. Some cancer patients have a genetic predisposition or other factors that make their blood more prone to clotting.

**Figure 1:**
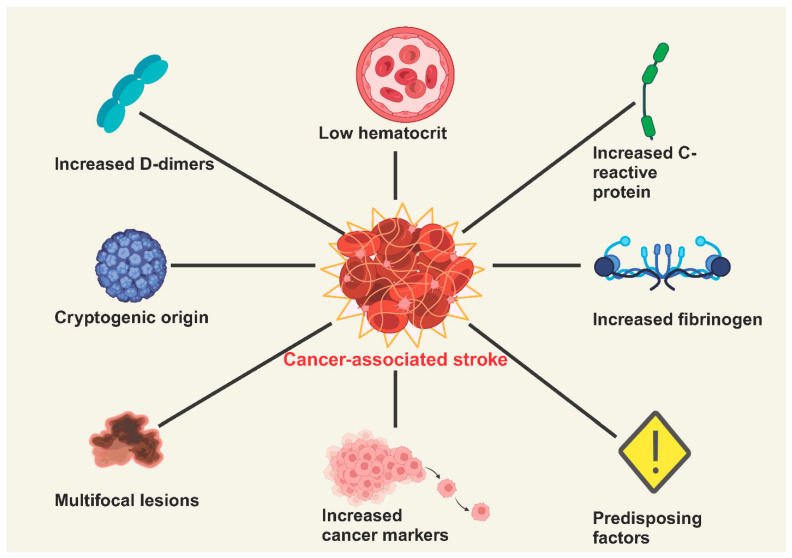
Factors affecting cancer-associated ischemic stroke.

These clottings can cause complications such as stroke, pulmonary embolism, or systemic arterial embolism. Hypercoagulability is one of the cancer-associated embolisms, where cancer cells produce procoagulant substances, such as tissue factor and cancer procoagulant, leading to clot formation. Systemic inflammation increases cytokine levels (e.g., IL-6, TNF-α), promoting coagulation and endothelial dysfunction. Direct embolization of tumor fragments or material (e.g., pieces of solid tumors or metastases) into the bloodstream can occur, particularly in cases of invasive or metastatic diseases. There is non-bacterial thrombotic endocarditis (NBTE), which is commonly associated with advanced cancers, especially adenocarcinomas. Sterile vegetation forms on heart valves, leading to arterial emboli. DVT or PE can lead to paradoxical embolism through a patent foramen ovale (PFO), causing strokes. The clinical manifestations of embolic stroke can be embolisms to other organs, such as the kidneys, spleen, or extremities (systemic arterial embolism), and they are often the result of venous thromboembolism secondary to malignancy like PE. Certain cancers are more likely to cause embolic events, including adenocarcinomas (e.g., pancreatic, gastric, lung), hematologic malignancies (e.g., multiple myeloma, lymphoma), and metastatic cancers [20,21,22] (Figure 1).

## 4. Stroke Patients and Cancer

A stroke could be the initial sign of a hidden cancer or may suggest a higher chance of developing cancer in the future. Cancer ranks second and stroke ranks fifth in terms of leading causes of death in the United States [23]. Both illnesses result in significant disability and economic burden on society. A study using *Nationwide Inpatient Sample* data found that 10% of ischemic stroke patients in hospitals also have cancer and suggests that this link could be increasing. Furthermore, within two years following an ischemic stroke, an additional 3–5% of patients are diagnosed with a new cancer [5]. Most comorbid cancers in stroke patients are solid tumors of the lung, gastrointestinal tract, and breast. Age- and sex-specific cumulative incidence are related to cancer patients in the years after a first-ever ischemic stroke. In addition, most patients with cancer and stroke are elderly and male, although patients of any age, sex, or race/ethnicity can be affected [24]. Patients aged 15 to 49 who have suffered a stroke may have a 3 to 5 times higher chance of developing cancer in the first year after the stroke compared to the general population. In contrast, individuals aged 50 or older have only a slightly increased risk. Among younger patients, the rate of new cancer following a stroke was higher in women compared to men. The rate of new cancer 10 years post-ischemic stroke was 3.7% for younger adults and 8.5% for older adults. Individuals who were younger adults had a 2.6 times higher chance of being diagnosed with a new cancer in the first year following an ischemic stroke compared to their counterparts in the general population [25]. Although some cancers of the gastrointestinal tract do not equally share smoking as a causative risk factor, most gastrointestinal and lung cancers are strongly associated with smoking, which is a widely accepted risk factor for stroke [26]. It was reported that younger adults have a higher chance of developing cancer within the first few years following an ischemic stroke or intracerebral hemorrhage, particularly in the first year, compared to their peers in the general population [25]. Increased risk of HIF-1α is associated with brain cancer among patients previously diagnosed with stroke, signifying that stroke might be seen as an early contributor and predictor of brain cancers [27], whereas cancer of the central nervous system, head and neck, lower respiratory, and urinary tract was more common in stroke patients compared to the general population [28]. The link between DVT and cancer is well established and stems from the complex interplay between tumor-related factors (TF, VEGF, IL-6, TNF-α), patient factors (hypercoagulability, immobilization, comorbidities), and treatment-related risks (surgery, chemotherapy, hormonal therapy, central venous catheters). The likelihood of cancer is increased for patients with DVT, particularly in the first six months following the occurrence. A study discovered that the likelihood of cancer was 1.3 times greater than anticipated for individuals with DVT. A different study revealed that 10–20% of individuals with DVT received a cancer diagnosis before or during the occurrence of the DVT [29,30].

## 5. Cancer Patients and Stroke

A total of 4.7% of cancer patients experience a stroke within 6 months of diagnosis, compared to 2.2% of those without cancer. The risk of fatal stroke is about twice as high for cancer patients than for the general population [31]. Among all cancer patients, the rate of stroke per 100,000 person-years was 21.64 [32]. The risk of having a stroke depends on the type of cancer, its histology, and its stage. Newly diagnosed solid or hematological cancers come with a significantly higher short-term risk of stroke. Pancreatic, gastric, and lung cancers, traditionally linked to venous thromboembolism, appear to pose the highest likelihood of arterial thromboembolism. In a *Medicare claims-based study*, it was found that 6.9% of elderly lung cancer patients had developed ischemic stroke one year after diagnosis, which was more than double the risk compared to 3.2% of matched controls. The risk of stroke is very closely linked to the stage of cancer, with the highest risks seen in stage 4 cancers, showing more than a ten-fold increase in risk in the initial month after cancer diagnosis [9,24]. The assessed incidence of stroke during the first year after an innovative diagnosis of cancer is 1.4%, with a higher risk for ischemic stroke [33]. This cross-sectional study found that the incidence of stroke among most age groups, head and neck cancer (HNC) subsites, stages, and treatment modalities in survivors of HNC was approximately 2.5 times that of the general population [34].

## 6. Types of Cancer and Stroke

CAS is more commonly associated with certain cancer types. The cancers most dominant in CAS include lung, pancreatic, gastrointestinal, and hematologic malignancies, with pancreatic and lung cancers showing the strongest associations due to their pronounced effects on coagulation and inflammation. The frequency of stroke in cancer patients primarily relies on the location of the cancer and stage of cancer (Table 1) [35]. Advanced cancer stages and metastatic disease further increase the risk of stroke. Among 1274 stroke patients admitted to a stroke unit, 12% received an additional cancer diagnosis, with urogenital, breast, and gastrointestinal cancers being the most common types [36]. One of the most frequent cancers associated with ischemic stroke is lung cancer [37]. Pancreatic cancer and gastrointestinal cancers are also strongly related to cancer-associated thrombosis, including ischemic stroke. Hematologic malignancies are associated with ischemic stroke, particularly in acute leukemias. Breast CAS is common in long-term cancer survivors with a risk of stroke. Primary or metastatic brain tumors (CNS tumors) can lead to ischemic stroke. Hypercoagulability, cancer stage, and therapies are important factors influencing cancer types in ischemic stroke. Understanding the cancer type is crucial for tailoring prevention and management strategies for CAS [17,24].

## 7. Extracellular Vesicles and Cancer-Associated Stroke

Extracellular vesicles (EVs) in circulation are involved in thrombosis linked to cancer and the advancement of tumors. Some propose that EVs from cancer cells contribute to coagulopathy, leading to ischemic stroke through mechanisms that do not involve tissue factor (TF) [40]. These vesicles, including exosomes, microvesicles, and apoptotic bodies, are released by cancer cells and stromal cells into the bloodstream, carrying various bioactive molecules. EVs also expose phosphatidylserine on their surface, which acts as a platform for coagulation enzyme complexes, amplifying thrombin generation and fibrin formation that contribute to VTE, ATE, and cancer-associated ischemic stroke. EVs interact with platelets and endothelial cells, inducing their activation and aggregation, further promoting clot formation and disrupting endothelial integrity by increasing vascular permeability. EVs also facilitate the formation of the pre-metastatic niche by altering the microenvironment, recruiting immune cells to support tumor cell colonization, and delivering oncogenic proteins, DNA, RNA, and microRNAs (e.g., miR-21, miR-122) that modulate recipient cell behavior to favor metastasis. Mice that received TF-positive EVs from monocytes exhibited heightened fibrin buildup, suggesting their role in arterial thrombosis. Utilizing low-TF mice, the origin of EVs responsible for arterial thrombosis was identified as neutrophil-derived TF-positive EVs attaching to endothelial cells through LFA-1 ICAM-1 interaction [41]. TF-positive EVs in venous thrombosis were examined across various models, showing a general correlation between TF and elevated EV levels along with an increase in thrombus mass. In individuals with the factor V Leiden mutation, circulating EVs, particularly TF-positive EVs, play a role in the onset of venous thrombosis [42,43]. Another predictor for cancer-associated thrombosis is an increased number of leukocytes by forming neutrophil extracellular traps (NETs). Neutrophils play an important role in thrombus development in cancer-associated thrombosis, and in a murine breast cancer model, it has been demonstrated that EVs released by tumor cells work together with neutrophils in venous thrombosis associated with cancer. Increased levels of circulating TF-positive EVs are associated with coagulation activation in vivo and increased thrombotic risk in human cancer patients [43,44]. Both plasmatic coagulation and platelet aggregation can be initiated or enhanced by EVs, thus playing a role in cancer-related thrombotic risks. Clinical trials targeting EV pathways in cancer and thrombosis are ongoing, showing promise for personalized medicine.

## 8. Inflammation and Cancer-Associated Ischemic Stroke

Inflammation in CAS plays a pivotal role in the development and progression of the condition. Cancer creates a pro-inflammatory state that disrupts vascular and hemostatic systems, increasing the risk of thromboembolic events, including ischemic strokes. The mechanisms linking inflammation and stroke in cancer involve a complex interplay of cellular and molecular processes that promote endothelial dysfunction, coagulopathy, platelet activation, and thrombus formation. These mechanisms lead to an increased risk of ischemic stroke and are influenced by tumor biology, the tumor microenvironment, and the systemic effects of cancer. Tumors secrete pro-inflammatory cytokines such as IL-1β, IL-6, TNF-α, and IL-8, which activate endothelial cells, increasing vascular permeability and procoagulant activity, producing more platelets, and enhancing the production of acute-phase reactants like C-reactive protein (CRP) and fibrinogen, both associated with thrombosis. IL-6, in particular, is linked to the upregulation of TF by stimulating platelet production via thrombopoiesis [45,46]. Increased CRP directly contributes to endothelial damage and thrombus formation. Those cytokines induce the expression of several adhesion molecules, such as intercellular adhesion molecule-1 (ICAM-1), vascular cell adhesion molecule-1 (VCAM-1), etc., on endothelial cells. Normal endothelium produces nitric oxide and prostacyclin to inhibit platelet aggregation and coagulation. Inflammatory mediators decrease their production, creating a prothrombotic environment, and downregulation of thrombomodulin further impairs anticoagulant activity [47]. Platelets bind to cancer cells via adhesion molecules, shielding tumor cells from immune attack, facilitating thrombus formation and further increasing the risk of embolic events. Tumor hypoxia increases the expression of hypoxia-inducible factors (HIFs), which stimulate angiogenesis and vascular remodeling, creating abnormal, leaky vessels prone to clot formation. Inflammation in cancer initiates and sustains a cascade of events, leading to vascular damage, coagulation activation, platelet aggregation, and thrombus formation, increasing the risk of ischemic stroke [48]. Understanding these mechanisms provides a basis for developing targeted therapies to reduce stroke risk in cancer patients. Further research into anti-inflammatory strategies and precision medicine approaches holds promise for improving outcomes in this high-risk population.

## 9. Atherosclerosis and Cancer-Associated Ischemic Stroke

Research has consistently been gathered to endorse a significant role for inflammation in the development of atherosclerosis and the risk of stroke in large arteries. Atherosclerosis is a major contributor to ischemic stroke in the general population and also plays a significant role in CAS. In cancer patients, atherosclerosis can interact with cancer-specific mechanisms, such as hypercoagulability, systemic inflammation, and treatment-related vascular damage, elevating stroke risk. Research findings from experimental studies suggest that inflammation plays a key role in every phase of the onset, advancement, degradation, and breakage of atherosclerotic plaque, resulting in thrombo-embolic occurrences [49]. Cancer induces systemic inflammation, mainly via IL-6 and TNF-α, which damages endothelial cells and increases the recruitment of inflammatory cells to atherosclerotic plaques. Some chemotherapeutic drugs such as cisplatin and 5-fluorouracil (5-FU) are associated with endothelial injury, oxidative stress, and atherosclerotic plaque progression. Radiation therapy or exposure in cancer patients causes radiation arteritis and accelerates atherosclerosis in large arteries like the carotid arteries, including fibrosis, plaque instability, and arterial narrowing. A significant number of cancer patients with ischemic stroke have evidence of large-artery atherosclerosis, such as carotid stenosis or intracranial atherosclerosis. Plaque rupture in carotid or vertebral arteries is a common source of embolic strokes too. Many CASs are classified as cryptogenic but may involve undetected atherosclerosis combined with cancer-related hypercoagulability or inflammation. Cancer-induced hypercoagulability amplifies thrombus formation at sites of plaque rupture, and *sterile vegetations* on heart valves can embolize in tandem with atherosclerotic plaque rupture, creating multiple embolic sources. Differentiating between stroke caused by large-artery atherosclerosis and other cancer-related mechanisms (e.g., non-bacterial thrombotic endocarditis (NBTE), hypercoagulability) can be challenging. Atherosclerosis in CAS exemplifies how traditional vascular risk factors intersect with cancer-specific mechanisms. Therefore, studies are needed to better understand the interaction between cancer-related inflammation, hypercoagulability, and atherosclerosis, and biomarker-driven approaches could improve the prediction of stroke risk in cancer patients with coexisting atherosclerosis.

## 10. Biomarkers and Cancer-Associated Ischemic Stroke

**Figure 2:**
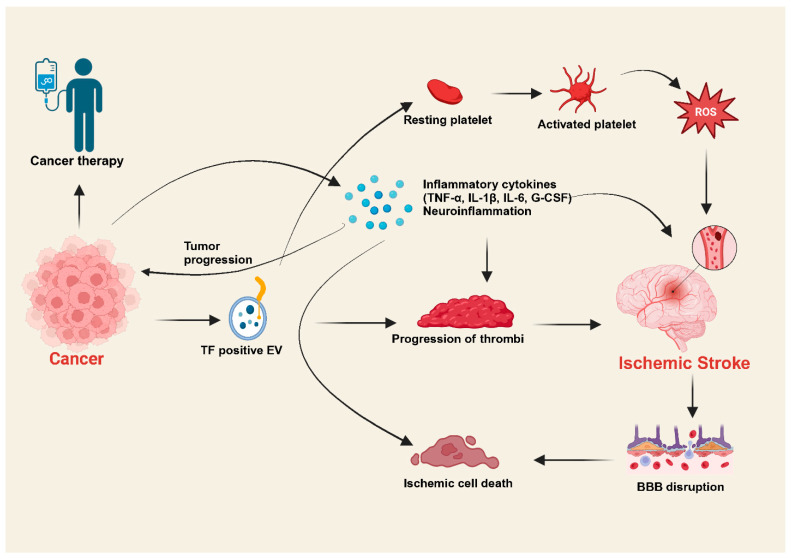
Potential mechanism of cancer-associated ischemic stroke. Cancer cells release inflammatory cytokines, TF-positive EVs, and neuroinflammatory molecules into the circulation. In addition, activated platelets induce ROS and mediate stroke. TF-positive EVs and inflammatory markers engage the progression of thrombi and ischemic stroke responsible for BBB disruption and ischemic cell death. EVs = extracellular vesicles; G-CSF = granulocyte colony-stimulating factor; BBB = blood-brain barrier; ROS = reactive oxygen species.

Biomarker panels can help identify patients at high risk for cancer-associated ischemic stroke. Key biomarkers linked to CAS are categorized by their relevance to inflammation, coagulation, endothelial dysfunction, and tumor activity. They can guide the effectiveness of anti-inflammatory and anticoagulant therapies. Key inflammatory biomarkers in CAS include CRP, IL-6, TNF-α, and NET. CRP and IL-6 are acute-phase reactants elevated during systemic inflammation and platelet activation, whereas TNF-α is involved in promoting endothelial dysfunction, leukocyte adhesion, and vascular inflammation. D-dimer, TF, and fibrinogen are coagulation and fibrinolysis biomarkers. They are involved in fibrin degradation, reflect clot formation, initiate the extrinsic coagulation pathway, and create fibrin in clot formation. Platelet factor 4 (PF4), soluble P-selectin, acts as a platelet activation biomarker. EVs (tumor-derived biomarkers), HIF-1α (hypoxia and angiogenesis biomarkers), and serum amyloid A (acute-phase reactants) are the foremost key biomarkers in CAS, which are involved in coagulation, inflammation, angiogenesis, vascular remodeling, and oxidative stress. Biomarkers were identified that had the potential to differentiate between patients who had cancer, stroke, or both conditions. It was shown that D-dimer was the most frequently monitored biomarker, and high levels were significantly associated with cancer-related strokes in 42/44 studies. A higher level of C-reactive protein, investigated in 19 studies, was also associated with cancer-related strokes, but a conclusive multivariate analysis was not performed. Fibrinogen was significantly associated with cancer-related strokes in 11/27 studies [50]. In addition to D dimer, CRP might serve as a potential biomarker for cancer-related strokes, but further clinical confirmation is needed. Fibrinogen, along with other biomarkers, has not been demonstrated to be useful in identifying CAS. These biomarkers provide essential insights into the pathophysiology of CAS and are valuable for risk stratification, diagnostic assessment, and monitoring therapeutic response (Figure 2) [50,51].

The Khorana and Vienna scores are valuable tools for assessing thrombotic risk in cancer patients, with potential applications in CAS. Tools like the Khorana score and Vienna score help identify high-risk patients who might benefit from prophylactic anticoagulation. The Khorana score predicts thrombotic events by identifying cancer patients at high risk of VTE. Since CAS is often embolic and linked to hypercoagulability, factors identified in the Khorana score may also correlate with stroke risk. Elevated biomarkers like platelet count and leukocyte count can indicate a heightened pro-thrombotic state by the Khorana score [52,53]. Alternatively, the Vienna score is a more recent risk assessment model specifically tailored for predicting thrombotic events in cancer patients. It integrates biomarkers, such as D-dimer and P-selectin, which are more directly associated with thrombotic risk, including stroke [54]. While the Khorana score provides a broad risk stratification framework, the Vienna score’s incorporation of biomarkers makes it particularly relevant to predicting stroke risk. However, neither score is validated specifically for arterial events like stroke, and their use in this context requires further research.

## 11. Transcription Factors and Cancer-Associated Ischemic Stroke

Transcription factors play a significant role in the pathogenesis of CAS by regulating genes involved in coagulation, endothelial dysfunction, inflammation, and tumor progression. Their dysregulated activity in cancer and vascular systems creates a pro-thrombotic and inflammatory environment conducive to stroke development. The master regulator of inflammation and coagulation in cancer is nuclear factor kappa B (NF-κB). It promotes the expression of pro-inflammatory cytokines (IL-6, IL-8) and chemokines by upregulating adhesion molecules (ICAM-1, VCAM-1) that recruit leukocytes to the endothelium and initiate the extrinsic coagulation cascade. HIF-1α is another key regulator, promoting angiogenesis and vascular remodeling, which upregulates glucose transporters and glycolytic enzymes to support tumor metabolism. Signal transducer and activator of transcription (STAT) 3 causes a hypercoagulable state and supports the formation of cancer-associated thrombi with vascular and thrombotic complications. Stress-responsive regulator activator protein-1 (AP-1) plays a significant role in this process by regulating the expression of matrix metalloproteinases (MMPs), leading to extracellular matrix degradation that contributes to plaque rupture and vascular remodeling, increasing ischemic stroke risk and endothelial cell apoptosis. E26 transformation-specific (ETS) transcription factors are responsible for the modulation of coagulation and angiogenesis. Some other important transcription factors that contribute to CAS are peroxisome proliferator-activated receptor (PPAR)-γ, forkhead box O (FOXO), Myc, and specific protein (Sp) 1 [55,56].

Kruppel-like factors (KLFs) are a family of zinc finger transcription factors that regulate critical cellular processes such as inflammation, endothelial function, angiogenesis, and thrombosis [57,58,59,60]. Their roles in CAS stem from their ability to influence both cancer progression and vascular health, particularly in the context of tumor-induced thromboembolism and vascular dysfunction. KLF2 and KLF4 are the protectors of endothelial integrity. Reduced levels lead to endothelial dysfunction, promoting thrombosis and vascular inflammation, key contributors to CAS. Increased KLF5 expression in cancer promotes endothelial cell activation and destabilization of atherosclerotic plaques, which can lead to ischemic events. It has been seen that mutations or downregulation of KLF6 in tumors may lead to enhanced tumor aggressiveness and pro-thrombotic states, increasing susceptibility to vascular damage and stroke. Dysregulated KLF10 in cancer patients may skew the transforming growth factor beta (TGF-β) signaling pathway toward pro-inflammatory effects, contributing to vascular inflammation and thrombotic events. Targeting KLF-related pathways offers a promising avenue for preventing and treating CAS, making them an important focus of translational research [61,62].

Overall, understanding the role of transcription factors in CAS provides valuable insights into the molecular mechanisms underpinning this condition. It also offers opportunities for developing targeted treatments to mitigate stroke risk in cancer patients.

## 12. Signaling Pathways and Cancer-Associated Ischemic Stroke

Signaling molecules and their pathways play pivotal roles in promoting inflammation, hypercoagulability, and vascular dysfunction [63]. They are related to a few prime signaling pathways such as inflammatory signaling pathways, IL-1β/NOD-like receptor protein 3 (NLRP3) inflammasome pathway, TNF-α/NF-κB pathway, TF pathway, platelet activation pathways, VEGF/angiogenesis pathway, EV pathway, HIF-1α pathway, and ICAM-1 and VCAM-1 pathway. IL-6 is a key pro-inflammatory molecule in this process, which binds to its receptor (IL-6R) on immune or endothelial cells and activates the Janus kinase (JAK) family of kinases and phosphorylates by activating STAT3 [64]. In the TNF-α/NF-κB pathway, activated TNF-α binds to the TNF receptor (TNFR), and NF-κB translocates to the nucleus to promote inflammatory gene expression [65]. However, NLRP3 inflammasome is activated by a tumor-derived signal in the immune cells that cleaves pro-IL-1β to active IL-1β by caspase-1 and finally promotes thrombosis in the IL-1β/NLRP3 pathway [66]. TF, NET, and platelet activation pathways are also important in coagulation and prothrombotic pathways for CAS mediated by TF, ROS, histones, thrombin, P-selectin, and ADP molecules. In the VEGF/angiogenesis pathway, hypoxic tumors secrete VEGF that activates phosphatidylinositol 3-kinase (PI3K)/protein kinase B (Akt) and mitogen-activated protein kinase (MAPK) pathways, leading to leaky and dysfunctional vasculature, contributing to embolism and stroke risk. Reactive oxygen species (ROS) production followed by an inflammation increase in endothelial cells is also initiated by tumor-induced hypoxia that promotes lipid oxidation, atherosclerosis, and vascular inflammation in the RO-mediated pathway. EV pathway also amplifies thrombosis and systemic inflammation by TF and microRNAs. Cytokines IL-6 and TNF-α are also involved in the upregulation of ICAM-1 and VCAM-1 on the endothelium, which enhances vascular inflammation and atherosclerosis. Understanding the signaling molecule pathways in CAS is of profound clinical and scientific importance [67,68,69]. These pathways reveal how cancer and stroke are interconnected through mechanisms involving inflammation, coagulation, and vascular dysfunction. By unraveling the intricate pathways linking cancer and stroke, clinicians and researchers can better diagnose, treat, and prevent CAS. This knowledge not only improves patient outcomes but also paves the way for breakthroughs in managing two of the most challenging medical conditions.

## 13. In Vivo Models of Cancer-Associated Ischemic Stroke

Animal models play a pivotal role in understanding the pathophysiology of CAS, exploring therapeutic targets, and identifying biomarkers. These models integrate cancer biology (e.g., tumor growth, hypercoagulability) with stroke induction to replicate the clinical condition seen in cancer patients. In vivo models for CAS combine tumor biology with stroke pathophysiology to explore mechanisms such as hypercoagulability, inflammation, and embolization. Models using tumor-derived EVs, cytokines, or metastatic-prone cell lines have enhanced our understanding of the disease. One study has assessed the impact of two distinct tumor types on ischemic stroke in mice. MC38 colon cancer cells or B16 melanoma cells were injected subcutaneously into C57BL/6 J mice, allowing tumors to grow before the mice underwent the permanent middle cerebral artery occlusion model. The mice with tumors exhibited enhanced neutrophil infiltration in the brain, heightened microglia activation, and elevated levels of IFN-γ and IL-1β. The mice with tumors exhibited greater neurological deficits [35,70]. However, each model has limitations, and a combination of models is often required to capture the full complexity of CAS. This was shown in a novel three-dimensional cancer-on-a-chip model by coculturing vessel-forming human umbilical vein endothelial cells with glioblastoma spheroids overexpressing TF, the initiator of coagulation for cancer-associated hypercoagulability [71]. In another study, mice were grafted with the aggressive breast cancer cell line MDA-MB-231 and injected with a synthetic small interfering (si) RNA targeting hepatic Serpinc1 to knockdown antithrombin to mimic a hypercoagulant state in vivo [72]. The transition from in vivo models (preclinical findings) to clinical patients’ implementation in CAS is a complex yet crucial process. These preclinical discoveries are being translated into clinical diagnostics, therapeutic strategies, and risk stratification models, bringing us closer to better management of stroke in cancer patients.

## 14. Diagnosis of Cancer-Associated Ischemic Stroke

In a clinical environment, vascular neurologists seek to identify the reason behind a stroke. When stroke patients exhibit several ischemic lesions on magnetic resonance imaging or elevated D-dimer levels without a clear reason for the stroke, occult cancer is suspected. In fact, acute stroke might be the initial clinical sign of hidden cancer. A Danish registry study revealed that numerous cancer locations are linked to an increased stroke risk because of hidden cancer. For example, the relative risk of ischemic stroke was higher in cancer patients than in the general population, with the following 95% confidence intervals: kidney cancer 2.42 (1.67–3.51), lung cancer 1.94 (1.68–2.23), and head and neck cancer 1.87 (1.29–2.7) [38,73].

## 15. Current Treatments for Cancer-Associated Ischemic Stroke

Despite the recent rise in publications regarding cancer-related stroke, evidence-based guidelines for its treatment are lacking. Similar to VTE, anticoagulation treatment is vital for patients experiencing both active cancer and ischemic stroke. Successful management of hypercoagulability contributes to the survival of patients with cancer-related strokes. Despite the ability of anticoagulant therapy to lower the chance of recurring thromboembolism, any decrease in this risk must be considered alongside the heightened risk of bleeding. Consequently, there is a pressing requirement for studies aimed at biologically unique, high-risk subgroups, which might benefit more from anticoagulant treatment. One specific subgroup is active cancer. In the prevention of CAS, regular screening and monitoring, anticoagulation therapy, antiplatelet therapy, and cancer therapy are important strategies. Low-molecular-weight heparin is popular in anticoagulation therapy, where it reduces the risk of thrombotic events, including ischemic strokes, especially in patients with advanced cancer, by preventing VTE. It is reported that long-term subcutaneous heparin treatment may prevent the recurrence or relapse of CAS [74]. Essential approaches to avert cancer-related stroke recurrence include rigorous management of modifiable risk factors such as blood pressure, cholesterol, and glucose levels, consistent surveillance for possible thrombotic incidents, compliance with recommended anticoagulant medications, management of cancer treatment side effects, and using rehabilitation and post-stroke care. Moreover, adopting a healthy lifestyle through a nutritious diet and routine physical activity and working closely with both an oncologist and neurologist to customize treatment according to the particular cancer type and stroke risk elements are important [75]. Some direct oral anticoagulants are another alternative anticoagulation therapy, especially for gastrointestinal and pancreatic cancers, though bleeding risks must be carefully managed. Aspirin is used to reduce arterial thrombotic events, especially in patients with atherosclerotic risk factors or mild thrombosis, as an antiplatelet therapy. Using anti-angiogenic agents by inhibiting VEGF (e.g., bevacizumab) reduces tumor vascularization and may help control abnormal clotting pathways and targeted therapies, and immunotherapy (e.g., checkpoint inhibitors) may reduce tumor burden, decreasing stroke risk indirectly. Regularly monitoring biomarkers and screening by imaging, controlling the risk of cardiovascular, and managing systemic inflammation driven by cancer or its treatments are important preventions for CAS [21,37].

Treating CAS involves managing the acute stroke event, addressing underlying cancer-related mechanisms, and preventing recurrence. Thrombolysis and mechanical thrombectomy are used within the therapeutic window for acute stroke management. Anti-inflammatory agents such as steroids or cytokine inhibitors (e.g., IL-6 inhibitors) are used to reduce inflammation and for stroke rehabilitation, and nutritional management is also used to improve recovery and general health [35]. These comprehensive approaches address both the acute and long-term management of CAS while considering the patient’s oncological and overall health status. Ongoing research into molecular mechanisms and personalized medicine will likely improve outcomes for these high-risk patients.

## 16. Conclusions

Cancer-associated ischemic stroke is a disease that is connected both epidemiologically and mechanistically, and its prevalence is expected to rise as cancer survival rates improve. Ischemic stroke is a frequent complication associated with cancer, exhibiting various mechanisms. Stroke related to cancer is linked to an increased risk of neurological decline and death, alongside a significant recurrence rate. Although there are many factors linked with CAS, we can bring forward potential variables: cancer site, advanced cancer stage/systemic metastasis, age, D-dimer, inflammatory molecules, platelets, neutrophils, and EVs. The secondary prevention of ischemic stroke associated with cancer is complicated, with minimal clinical trial proof available to direct the choice of antiplatelet treatment and anticoagulants. Instead of conducting general studies on patients with CAS, future research should concentrate on those with ischemic stroke linked to cancer to develop optimal treatment options. Since cancer is a diverse and evolving disease with distinct medical and psychological challenges, neurologists and oncologists need to collaborate closely and adopt a patient-focused and holistic strategy to effectively manage these individuals. There is an urgent need for the advancement of science for the development of effective prevention and treatment methods for CAS. Creating a risk assessment tool to pinpoint cancer patients with a high likelihood of stroke would be extremely advantageous.

## 17. Future Directions

Additional findings are warranted that aim to develop standardized guidelines for risk assessment and treatment of stroke related to cancer. Considering that various mechanisms lead to ischemic cerebrovascular disease, personalized treatment strategies grounded in molecular and transcriptional characteristics are essential.

## Figures and Tables

**Table 1:** Occurrence of stroke in cancer types [38,39].

Site of Cancer	1-Year Cumulative Incidence of Stroke (%)	Altered Hazard Ratio
Pancreatic	1.52	High
Brain	1.71	High
Lung	1.57	High
Liver	0.97	Medium
Renal	1.16	Medium
Esophageal	1.57	Medium
Rectal	1.56	Medium
Stomach	1.52	Medium
Colon	1.58	Medium
Bladder	1.82	Low
Hematological	0.98	Low
Prostate	1.17	Low
Stomach	1.52	Low
Gynecological	0.90	Low
Breast	0.63	Low

## Data Availability

Not applicable.
